# Efficacy of a web- and text messaging-based intervention to reduce problem drinking in young people: study protocol of a cluster-randomised controlled trial

**DOI:** 10.1186/1471-2458-14-809

**Published:** 2014-08-07

**Authors:** Severin Haug, Tobias Kowatsch, Raquel Paz Castro, Andreas Filler, Michael P Schaub

**Affiliations:** Swiss Research Institute for Public Health and Addiction at Zurich University, Konradstrasse 32, Zurich, 8031 Switzerland; Health-IS Lab, Institute of Technology Management, University of St. Gallen, Dufourstrasse 40a, St. Gallen, 9000 Switzerland; Health-IS Lab, Chair of Information Management, ETH Zurich, Weinbergstrasse 56/58, Zurich, 8092 Switzerland; Trier University of Applied Sciences, Environmental Campus Birkenfeld, P.O. Box 1380, Birkenfeld, 55761 Germany

**Keywords:** Alcohol, Problem drinking, Web, Internet, Text messaging, Social norms, Young people, Students

## Abstract

**Background:**

Problem drinking, particularly risky single-occasion drinking is widespread among adolescents and young adults in most Western countries. Mobile phone text messaging allows a proactive and cost-effective delivery of short messages at any time and place and allows the delivery of individualised information at times when young people typically drink alcohol. The main objective of the planned study is to test the efficacy of a combined web- and text messaging-based intervention to reduce problem drinking in young people with heterogeneous educational level.

**Methods/Design:**

A two-arm cluster-randomised controlled trial with one follow-up assessment after 6 months will be conducted to test the efficacy of the intervention in comparison to assessment only. The fully-automated intervention program will provide an online feedback based on the social norms approach as well as individually tailored mobile phone text messages to stimulate (1) positive outcome expectations to drink within low-risk limits, (2) self-efficacy to resist alcohol and (3) planning processes to translate intentions to resist alcohol into action. Program participants will receive up to two weekly text messages over a time period of 3 months. Study participants will be 934 students from approximately 93 upper secondary and vocational schools in Switzerland. Main outcome criterion will be risky single-occasion drinking in the past 30 days preceding the follow-up assessment.

**Discussion:**

This is the first study testing the efficacy of a combined web- and text messaging-based intervention to reduce problem drinking in young people. Given that this intervention approach proves to be effective, it could be easily implemented in various settings, and it could reach large numbers of young people in a cost-effective way.

**Trial registration:**

Current Controlled Trials ISRCTN59944705.

## Background

Alcohol use is a major cause of the disease burden in most countries of the world [1]. In adolescents and young adults of developed countries, alcohol use constitutes the greatest risk factor for mortality and morbidity [2]. Problem drinking is associated with multiple social and interpersonal problems such as arguing with friends and parents, engaging in unplanned sexual activity, drinking and driving, assault, getting into trouble with the law, academic difficulties, unintended injuries, and suicidal acts. In the long term, problem drinkers have an elevated risk of developing chronic diseases, e.g., heart and liver diseases or alcohol dependence [3–5].

Indicators of problem drinking are (1) a daily average consumption of three or more standard drinks for men, and two or more for women [6] and (2) risky single-occasion drinking (RSOD, also called binge drinking), defined as drinking 5 or more drinks on an occasion for men and 4 or more drinks for women [7]. The prevalence rates of RSOD are particularly high in adolescence and young adulthood [8]. In Switzerland, the prevalence of at least monthly RSOD is 28% in adolescents ages 15 to 19 and 42% in young adults ages 20 to 24 [9]. Compared to RSOD, the prevalence of an increased mean daily consumption in adolescence and young adulthood is relatively low (2% at ages 15 to 19 and 3% at ages 20 to 24) and it always occurs in combination with RSOD [9].

Studies testing the efficacy of interventions to reduce problem drinking among adolescents were predominantly conducted in non-European countries and were targeted to college or university students [10]. Within this target group, individual interventions using motivational interviewing [11] or personalized normative feedback based on the social norms approach [12] were effective to reduce alcohol consumption and alcohol related problems [13].

While interventions based on motivational interviewing are typically provided in face-to-face counselling sessions, social norms interventions were mostly provided via computer generated tailored letters or web-feedback. Social norm refers to our perceptions and beliefs on what is “appropriate” or “normal” behaviour of people close to us. Alcohol use misperceptions, i.e. overestimations of the amount of alcohol consumed, have been found in several studies, primarily in samples of adolescents with higher educational background [12] but also in young people with heterogeneous educational levels [14]. The presentation of correct information about peer group drinking norms in a credible way is hypothesized to reduce perceived peer pressure for high levels of alcohol consumption in both problem drinkers and non-drinkers [12]. The effectiveness of social norms interventions to reduce alcohol misuse in university and college students was analysed in a Cochrane review [15]. This review concluded that normative feedback interventions delivered using the web or computer reduced drinking quantity, drinking frequency, and binge drinking in the short- and medium term.

Social norm interventions to reduce problem drinking typically consist of a single intervention session in which participants receive tailored web or printed feedback. Due to their length of up to 7–8 pages of text and graphics, these feedbacks are primarily suitable for individuals with higher educational level. To provide shorter and more recurrent feedback messages might not only be a more effective approach particularly for individuals with lower educational levels but also to support and maintain behaviour change over a longer time period. Mobile phone text messaging provides a suitable technology to deliver short and repeated messages. This service allows a cost-effective instantaneous delivery of short messages directly to individuals at any time and place. In the field of alcohol prevention, text messaging particularly allows the delivery of individualized messages at times when young people typically drink alcohol [16]. In Switzerland, as in most other developed countries, nearly all adolescents (95%) between the ages of 12 and 19 own a mobile phone and text messaging is the most commonly used mobile phone application [17].

Mobile phone text messaging is increasingly applied in the context of behaviour change interventions, particularly for smoking cessation and diabetes self-management [18, 19]. For alcohol treatment, two pilot studies based on small sample sizes are available. Suffoletto et al. [20] reported fewer heavy drinking days and fewer drinks per drinking day in 15 young adults reporting harmful alcohol use who received text messages supporting goal setting up to 3 months after emergency treatment. In a study using twice daily supportive text messages (n = 26) or a fortnightly thank you text message (n = 28) for 3 months, co-morbid depressive and alcohol dependent patients reported lower depression scores and a trend for higher cumulative abstinence duration [21].

Within a pre-post-study, the acceptance and initial efficacy of a combined, individually tailored web- and text messaging-based intervention program to reduce problem drinking in young people with predominantly lower educational level was tested [22]. This fully automated program provided (1) an online feedback about an individual’s drinking pattern compared to the drinking norms of an age- and gender-specific reference group and (2) recurrent individualized text messages over a time period of 3 months. The program was tested in 36 school classes at 7 vocational schools in Switzerland. Irrespective of their drinking behaviour, 477 apprentices who owned a mobile phone were invited to participate in the program. Of these, 364 (76%) participated in the program. The percentage of persons with RSOD significantly decreased from 76% at baseline to 68% at the 3-month follow-up assessment. Furthermore, a significant decrease in the percentage of persons with alcohol-related problems and in the mean number of standard drinks per week could be observed. Within this study protocol, we describe a randomised controlled trial testing the efficacy of an optimized version of this program, called *MobileCoach Alcohol,* within a controlled trial.

## Methods/design

### Design and hypotheses

**Figure 1:**
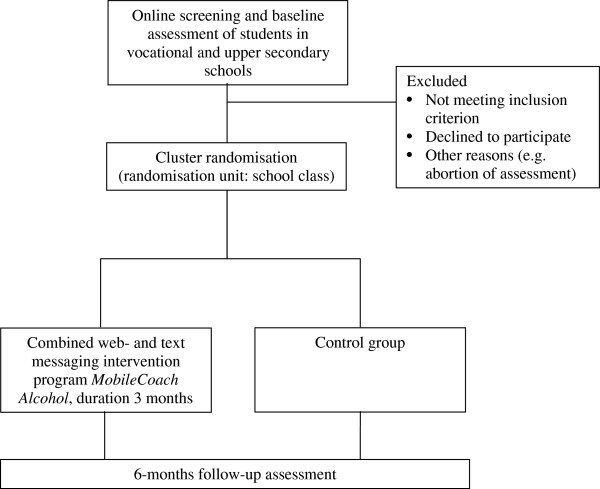
**Study design.**

A two-arm cluster-randomised controlled trial will be conducted to test the efficacy of the *MobileCoach Alcohol,* a combined web- and text messaging-based intervention to reduce problem drinking in young people. The efficacy of the intervention will be tested in comparison to an assessment only control group. The study participants will be assessed at baseline and at 6-month follow-up (Figure 1).

### Participants, setting and procedure

The *MobileCoach Alcohol* will be tested in vocational and upper secondary school students due to (1) their heterogeneous educational level and (2) their age range, primarily between 16 and 20 years of age. In this age group, alcohol consumption increases considerably compared to younger age [9] and the use of Internet and mobile phone text messaging is widespread [17]. Vocational and upper secondary schools in the Swiss Cantons of Zurich and Berne will be invited by prevention specialist centres in Zurich and Berne to participate in the study.

The vocational and upper secondary school students will be invited for study participation during a regular school lesson reserved for health education. They will be invited by junior scientists to participate in a study testing innovative channels of information about alcohol consumption. To ensure sufficient participation and, thus, representativeness of the sample [23], a reward of 10 Swiss Francs for participation in the study at both baseline- and follow-up assessment will be announced. Each student will receive a tablet computer for conducting the screening, baseline assessment, and study registration. Inclusion criterion for study participation is possession of a mobile phone. After receiving informed consent, study participants will be invited to choose a username and to provide their mobile phone number. Participants of the intervention group will receive additional questions that are necessary for tailoring the intervention content. Subsequently, participants in the intervention group will receive an individualised feedback based on the social norms approach on their tablet computer. During the subsequent 3 months, participants of the intervention group will receive one or two individually tailored text messages per week to reduce problem drinking. Participants of the assessment-only group will receive no intervention. Computer-assisted follow-up assessments after 6 months will be conducted by a junior scientist, within the participating school classes, during regular school lessons and using tablet computers.

### Ethical review

The study protocol was approved by the Ethics Committee of the Philosophical Faculty of the University of Zurich, Switzerland (date of approval June, 24^th^, 2014). The trial will be executed in compliance with the Helsinki Declaration.

### Randomisation and allocation concealment

To avoid spill-over effects within school classes, we will conduct a cluster-randomised controlled trial using school class as a randomisation unit. Due to the heterogeneity of apprentices in the different vocational and upper secondary school classes (e.g. concerning gender or professions), we will use separate randomisation lists for each school (stratified randomisation). Furthermore, to approximate equality of sample sizes in the study groups, we will use block randomisation with computer generated randomly permuted blocks of 4 cases [24].

The junior scientists supervising the baseline assessment in the vocational schools will be blinded concerning group allocation of the school classes. Also, group allocation will not be released to study participants until having provided informed consent, their username, mobile phone number, and baseline data. Furthermore, the junior scientists conducting the computer assisted follow-up assessments will be blinded when assessing the primary and secondary outcome measures.

### Sample size calculation

An estimation of the expectable effect size was based on the results of the pre-post study testing the initial efficacy of the program [22]. This study revealed a decrease in the percentage of persons with at least one RSOD occasion in the last month from 76% at baseline assessment to 68% at follow-up assessment. It is assumed that the improvements in the content and tailoring of the intervention will also result in an improved efficacy. Based on these considerations, it is assumed that the percentage of persons with at least one RSOD occasion in the last month at follow-up will be 76% in the control group and 66% or lower in the intervention group. A sample size of n = 322 in each study group would be required to have 80% power for a χ^2^-test (α = 5%, 2-sided) in order to detect this difference based on a calculation using *G-Power* [25]. As students are nested within school classes, a potential design effect needed to be considered. Based on [22] an average cluster size of 10 study participants per school class and an intra-cluster correlation coefficient of 0.05 could be expected. This would result in a design effect of 1.45. Multiplying this design effect by the required size for an unnested sample (n = 322) results in a required sample size of n = 467 per study group and a total of n = 934 study participants.

### Intervention

#### Technological background

The *MobileCoach Alcohol* was developed on the *MobileCoach* system. This is a modular system that allows an author to easily modify and optimize the single modules without any technical programming skills: e.g., the baseline assessment survey, the tailored web-based feedback, the intervention rules and content elements. The source code of the *MobileCoach* system will be made available as open source project on http://mobile-coach.eu by the end of 2014.

Building on the foundations of automata theory [26], the technical design of the *MobileCoach* system follows the concepts of a state machine that uses intervention rules for state transitions, which can be referred to as a fully automated expert system. Here, the state is an aggregate of all relevant attributes related to the intervention progress of a participant (e.g. the messages received or answers provided) whereas state transitions triggered by intervention rules lead to a change in these attributes and thus, to a state change. In particular, each participant of the intervention group is assigned to a particular intervention state based on her answers during the baseline assessment. In response to this assessment, a web-based feedback is generated individually by the system for each participant. Then, depending on a participant’s regular text message feedback during the subsequent 3 months, intervention rules trigger state transitions and the tailoring of the follow-up text messages. In particular, intervention rules are traversed once a day for each participant, and, as a result, update the state of the corresponding participant and fire text messages in the form of a question, a feedback text or a recommendation.

The technical architecture of this rule-based state machine is derived from the *model-view-controller* design pattern [27]. It consists therefore of (1) a persistence layer, i.e. the *model* based on the document database mongoDB (https://www.mongodb.org) and plain files for storing the intervention content including a detailed protocol of all incoming and outgoing text messages, (2) an application layer, i.e. the *view* based on the Vaadin web application framework (http://www.vaadin.com) with the template-engine Mustache (http://mustache.github.io) for intervention administration and the assessment survey at the baseline and, finally (3) a service layer, i.e. the primary controller that utilizes the Java programming language (http://java.com) and the expression evaluator Javaluator (http://javaluator.sourceforge.net) for the evaluation of the intervention rules. Password protection and Secure Sockets Layer (SSL) encoding will be used to ensure the privacy and safety of data transfer.

#### Theoretical background

The web-based part of the intervention provides normative feedback based on the social norms approach [12], which constitutes the theoretical background of the majority of evidence based internet interventions to reduce problem drinking in young people [15, 28].

The text messaging based part of the intervention also includes elements of the social norms approach (e.g., individual risk patterns of alcohol related negative consequences). However, it primarily relies on socio-cognitive constructs from major psychological models of health behaviour change, such as the Social Cognitive Theory [29] and the Health Action Process Approach [30]: outcome expectations, self-efficacy and planning processes. Within both models, positive outcome expectations, e.g., beliefs about the likelihood and value of the consequences of drinking less alcohol, substantially contribute to forming an intention to perform a desired action. Furthermore, self-efficacy, e.g., beliefs about the personal ability to resist alcohol in different drinking situations, is crucial in forming an intention to perform a desired action. Planning processes such as if-then plans that link situational cues with responses that are effective in attaining a desired outcome [31] are seen as particularly relevant to bridge the intention-behaviour gap and to translate behavioural intentions into actions.

#### Web-based feedback

The web-based feedback was already developed for a previous study [22]. Its interventional content is based on effective social norms intervention programs developed primarily for college and university students in the USA and Canada [32, 33] that had been modified for the target group of German-speaking adolescents in Switzerland aged 16 to 20 with different educational backgrounds. Age- and gender-specific norms for alcohol consumption were derived from a previous study [34] that assessed heavy drinking occasions, alcohol volume and the maximum number of drinks on a single occasion among 973 vocational and upper secondary school students in the Canton of Zurich, Switzerland. The web-based feedback includes individually tailored graphical and textual information concerning (1) the number of drinks consumed per week in relation to the age and gender-specific reference group (2) financial costs of drinking, (3) calories consumed with alcoholic drinks and (4) number of RSOD occasions in relation to the age and gender-specific reference group.

#### Text messages

On the first level, the content and number of the provided text messages will be tailored according to baseline drinking patterns. Participants will be assigned to one of three risk groups: (1) “Low-Risk”: no RSOD occasion during the last 30 days; (2) “Medium-Risk”: 1 or 2 RSOD occasions during the last 30 days and (3) “High Risk”: > 2 RSOD occasions during the last 30 days.

On the second level, the content of the text messages will be tailored according to the individual values on the following baseline variables: sex, motivation for reduced alcohol consumption, alcohol-related problems, typical drinking day and time, peak blood alcohol concentration during the previous 30 days, positive outcome expectancies, typical drinking situations, strategies to resist alcohol in different drinking situations, place of assessment (Canton Zurich vs. Canton Berne). Participants from all risk groups will receive text messages for a period of 3 months.

Participants of the low-risk group will receive one weekly text message providing information from the following content categories:

1. Motivation for drinking within low-risk limits using individual data on positive outcome expectancies derived from [35].
2. Strategies to resist alcohol in different drinking situations using individual data from the adolescent version of the Drinking Refusal Self-Efficacy Questionnaire (DRSEQ-RA) [36].

Participants of the medium-risk group will receive two weekly text messages. One weekly text message will be sent on a fixed weekday in the afternoon. It will provide information from one of the following content categories:

1. Motivation for drinking within low-risk limits using individual data on positive outcome expectancies derived from a list provided by [35].
2. Alcohol-related problems using individual data on previous alcohol-related problems.
3. Peak blood alcohol concentration and related risks using data on sex, body weight and maximum number of drinks on a single occasion in the previous month.

Additionally, they will receive one weekly text message sent on the individually indicated typical drinking day and time. This text message focuses on the following aspects:

4. Strategies to resist alcohol in different drinking situations using data on individual drinking situations and individual chosen strategies to resist alcohol. Participants will be asked at baseline to select 3 among 9 provided drinking situations, derived from [36], that are chosen as most tempting. For each of the 3 chosen drinking situations, they can choose 1 out of 3 strategies to resist alcohol. The resulting strategies are “if-then” plans based on the concept of implementation intentions [31], e.g., “When I am at a party, I will keep track of the amount I drink. I decide how much I will drink ahead of time and stick to this limit”.

Like participants of the medium-risk group, participants of the high-risk group will also receive two weekly text messages from the content categories (1) – (4). Additionally, they will receive information about local outpatient services for alcohol counselling using data on the place of assessment. Sample text messages for different risk groups and content categories are shown in Table 1.

**Table 1:** **Sample text messages derived from individual data**

Risk group	Content category	Considered individual data	Resulting text message
Low-risk	Motivation for drinking within low-risk limits	Indicated “Yes” for the item: “If I drink within low-risk limits, other people will respect me”.	Hi Lisa. Great! You are not just a follower who drinks alcohol to fit in. That’s a smart decision. It shows strength of character and can even impress others.
Medium-risk	Strategies to resist alcohol in different drinking situations	Individually chosen if-then plan to resist alcohol in the tempting drinking situation “party”: “When I am at a party, I have soft drinks every now and then”.	Hey Luca. Congratulations! It’s a good decision to have soft drinks every now and then, when you are at a party! Non-alcoholic drinks provide your body with important minerals and are a thirst-quenching alternative.
High-risk	Local outpatient services for alcohol counselling	Place of assessment: Zurich	Hi Robin. Are you concerned about your own alcohol intake or that of a friend? Talking to someone about it can be really helpful. The website http://www.alcocheck.ch can offer you support. Write an e-mail to info@alcocheck.ch or call 044 444 77.

### Assessments and outcomes

At baseline, demographic variables (age, gender, educational level, and migration background) as well as characteristics of the schools and school classes will be assessed.

Baseline- and follow-up assessments will include:

1. Frequency of RSOD occasions in the last 30 days (“How often did you have 5 (male; female: 4) or more drinks on one occasion in the last 30 days?”).
2. Quantity of alcohol consumption, assessed by a 7-day drinking calendar similar to the Daily Drinking Questionnaire (DDQ) [37], for which participants were asked to think about a typical week in the past month and, for each day, to record the number of standard drinks they typically consumed on that day.
3. Peak blood alcohol concentration assessed by asking participants to report the number of standard drinks consumed and the duration of their heaviest drinking episode in the previous 30 days. This information will be used along with the sex and weight to calculate an estimated peak blood alcohol concentration based on the Widmark Formula [38].
4. Normative misperceptions of alcohol consumption using reference data from [39] and items derived from [14], who used modified versions of the first and second AUDIT-C [14, 40] items: “How often does a typical (male/female) secondary school student have a drink containing alcohol?” and “How many drinks does a typical (male/female) secondary school student have on a typical day when drinking alcohol?”.

The primary outcome of the planned study is RSOD in the past 30 days preceding the follow-up assessment. Secondary outcomes are (1) frequency of RSOD occasions in the past 30 days preceding the follow-up assessment, (2) peak blood alcohol concentration in the previous 30 days, (3) number of standard drinks consumed in a typical week of the preceding month, and (4) normative misperceptions of alcohol consumption in an age- and gender-specific reference group.

### Data analyses

Generalized Estimation Equation analyses (GEE) [41] will be used to test the efficacy of the intervention on the different outcome measures. Based on the scale level of the measurement and its distribution, logistic GEE-models, linear GEE-models or GEE-models for count variables will be applied. Potential baseline differences will be considered by adding additional baseline variables as covariates to the GEE-models. Both (1) complete case analyses (CCA) considering all study participants with available follow-up data and (2) intention to treat (ITT) analyses will be conducted. For the ITT analyses, the multiple imputations procedure (MICE) of STATA will be applied. Given the clustered nature of the data, robust variance estimators for all GEE-models will be computed, using the cluster option of STATA.

## Discussion

This study protocol presents the design of a cluster randomised controlled trial testing the efficacy of a combined web- and text messaging-based intervention to reduce problem drinking in young people. Although web-based interventions have been applied and tested for nearly all modifiable and preventable risk factors [42–44], the efficacy of mobile phone text messaging or combined web- and text messaging-based interventions has not received much scientific attention so far. This is the first study testing the efficacy of a combined web- and text messaging-based intervention to reduce problem drinking in young people and one of the few studies testing the efficacy of a web-based intervention to reduce problem drinking in young people with heterogeneous educational level. In contrast to individual face-to-face counselling, counselling via Internet and text messaging is more economic and matches with the lifestyle and communication habits of young people. Given that this intervention approach proves to be effective, it could be disseminated to various groups of adolescents and young adults, e.g. in schools or at workplaces.

## Abbreviations

RSOD: Risky single-occasion drinking.

## References

[1] Lim SS, Vos T, Flaxman AD, Danaei G, Shibuya K, Adair-Rohani H, Amann M, Anderson HR, Andrews KG, Aryee M, Atkinson C, Bacchus LJ, Bahalim AN, Balakrishnan K, Balmes J, Barker-Collo S, Baxter A, Bell ML, Blore JD, Blyth F, Bonner C, Borges G, Bourne R, Boussinesq M, Brauer M, Brooks P, Bruce NG, Brunekreef B, Bryan-Hancock C, Bucello C (2012). A comparative risk assessment of burden of disease and injury attributable to 67 risk factors and risk factor clusters in 21 regions, 1990–2010: a systematic analysis for the global burden of disease study 2010. Lancet.

[2] Rehm J, Taylor B, Room R (2006). Global burden of disease from alcohol, illicit drugs and tobacco. Drug and Alcohol Review.

[3] Hingson RW, Heeren T, Edwards EM (2008). Age at drinking onset, alcohol dependence, and their relation to drug use and dependence, driving under the influence of drugs, and motor-vehicle crash involvement because of drugs. Journal of Studies on Alcohol and Drugs.

[4] Hingson RW, Edwards EM, Heeren T, Rosenbloom D (2009). Age of drinking onset and injuries, motor vehicle crashes, and physical fights after drinking and when not drinking. Alcoholism: Clinical and Experimental Research.

[5] Anderson P, Baumberg B (2006). Alcohol in Europe: a public health perspective: a report for the European commission.

[6] British Medical Association (BMA) (1995). Alcohol: guidelines on sensible drinking.

[7] Gmel G, Kuntsche E, Rehm J (2011). Risky single-occasion drinking: bingeing is not bingeing. Addiction.

[8] Kuntsche E, Rehm J, Gmel G (2004). Characteristics of binge drinkers in Europe. Social Science and Medicine.

[9] Gmel G, Kuendig H, Notari L, Gmel C, Flury R (2013). Addiction monitoring in Switzerland – data 2012.

[10] Bewick BM, Trusler K, Barkham M, Hill AJ, Cahill J, Mulhern B (2008). The effectiveness of web-based interventions designed to decrease alcohol consumption - a systematic review. Preventive Medicine.

[11] Miller JH, Moyers T (2002). Motivational interviewing in substance abuse: applications for occupational medicine. Occupational Medicine.

[12] Perkins HW (2003). The social norms approach to preventing school and college age substance abuse: a handbook for educators, counselors, and clinicians, first edn.

[13] Carey KB, Scott-Sheldon LA, Carey MP, DeMartini KS (2007). Individual-level interventions to reduce college student drinking: a meta-analytic review. Addictive Behaviors.

[14] Haug S, Ulbricht S, Hanke M, Meyer C, John U (2011). Overestimation of drinking norms and its association with alcohol consumption in apprentices. Alcohol and Alcoholism.

[15] Moreira MT, Smith LA, Foxcroft D (2009). Social norms interventions to reduce alcohol misuse in university or college students. Cochrane Database of Systematic Reviews.

[16] Kuntsche E, Robert B (2009). Short message service (SMS) technology in alcohol research–a feasibility study. Alcohol and Alcoholism.

[17] Willemse I, Waller G, Süss D, Genner S, Huber A-L (2012). JAMES: Jugend, Aktivitäten, Medien - Erhebung Schweiz: Zürich: Zürcher Hochschule für angewandte Wissenschaften.

[18] Whittaker R, McRobbie H, Bullen C, Borland R, Rodgers A, Gu Y (2012). Mobile phone-based interventions for smoking cessation. Cochrane Database of Systematic Reviews.

[19] Fjeldsoe BS, Marshall AL, Miller YD (2009). Behavior change interventions delivered by mobile telephone short-message service. American Journal of Preventive Medicine.

[20] Suffoletto B, Callaway C, Kristan J, Kraemer K, Clark DB (2012). Text-message-based drinking assessments and brief interventions for young adults discharged from the emergency department. Alcoholism: Clinical and Experimental Research.

[21] Agyapong VI, Ahern S, McLoughlin DM, Farren CK (2012). Supportive text messaging for depression and comorbid alcohol use disorder: single-blind randomised trial. Journal of Affective Disorders.

[22] Haug S, Schaub MP, Venzin V, Meyer C, John U, Gmel G (2013). A pre-post study on the appropriateness and effectiveness of a web- and text messaging-based intervention to reduce problem drinking in emerging adults. Journal of Medical Internet Research.

[23] Edwards P, Cooper R, Roberts I, Frost C (2005). Meta-analysis of randomised trials of monetary incentives and response to mailed questionnaires. Journal of Epidemiology and Community Health.

[24] Pocock SJ (1994). Clinical trials: a practical approach.

[25] Faul F, Erdfelder E, Lang AG, Buchner A (2007). G*Power 3: a flexible statistical power analysis program for the social, behavioral, and biomedical sciences. Behavior Research Methods.

[26] Hopcroft JE, Motwani R, Ullman JD (2013). Introduction to automata theory, languages, and computation.

[27] Gamma E, Helm R, Johnson RE, Vlissides J (1994). Design patterns: elements of reusable object-oriented software.

[28] Haug S, Sannemann J, Meyer C, John U (2012). Reduktion des Alkoholkonsums und Förderung der Rauchabstinenz bei Jugendlichen und jungen Erwachsenen mittels Internet und Mobiltelefon: ein Literaturüberblick. [internet and mobile phone interventions to decrease alcohol consumption and to support smoking cessation in adolescents: a review]. Gesundheitswesen.

[29] McAlister AL, Perry CL, Parcel GS, Glanz K, Rimer BK, Viswanath K (2008). How individuals, environments, and health behaviour interact: social cognitive theory. Health behavior and health education: theory, research, and practice.

[30] Schwarzer R (2008). Modeling health behavior change: how to predict and modify the adoption and maintenance of health behaviors. Applied Psychology: An International Review.

[31] Gollwitzer PM, Sheeran P (2006). Implementation intentions and goal achievement: a meta-analysis of effects and processes. Advances in Experimental Social Psychology.

[32] Cunningham JA, Humphreys K, Kypri K, van Mierlo T (2006). Formative evaluation and three-month follow-up of an online personalized assessment feedback intervention for problem drinkers. Journal of Medical Internet Research.

[33] Doumas DM, McKinley LL, Book P (2009). Evaluation of two web-based alcohol interventions for mandated college students. Journal of Substance Abuse Treatment.

[34] Gmel G, Venzin V, Marmet K, Danko G, Labhart F (2012). A quasi-randomized group trial of a brief alcohol intervention on risky single occasion drinking among secondary school students. International Journal of Public Health.

[35] Babor TF, Higgins-Biddle JC (2001). Brief intervention for hazardous and harmful drinking: a manual for use in primary care.

[36] Young RM, Hasking PA, Oei TP, Loveday W (2007). Validation of the drinking refusal self-efficacy questionnaire - revised in an adolescent sample (DRSEQ-RA). Addictive Behaviors.

[37] Collins RL, Parks GA, Marlatt GA (1985). Social determinants of alcohol consumption: the effects of social interaction and model status on the self-administration of alcohol. Journal of Consulting and Clinical Psychology.

[38] Yang CT, Fung WK, Tam TW (2009). Alcohol study on blood concentration estimation: reliability and applicability of Widmark formula on Chinese male population. Legal Medicine.

[39] Haug S, Schaub M, Salis Gross C, John U, Meyer C (2013). Predictors of hazardous drinking, tobacco smoking and physical inactivity in vocational school students. BMC Public Health.

[40] Bradley KA, DeBenedetti AF, Volk RJ, Williams EC, Frank D, Kivlahan DR (2007). AUDIT-C as a brief screen for alcohol misuse in primary care. Alcoholism: Clinical and Experimental Research.

[41] Zeger SL, Liang KY, Albert PS (1988). Models for longitudinal data: a generalized estimating equation approach. Biometrics.

[42] Davies CA, Spence JC, Vandelanotte C, Caperchione CM, Mummery WK (2012). Meta-analysis of internet-delivered interventions to increase physical activity levels. The International Journal of Behavioral Nutrition and Physical Activity.

[43] Rooke S, Thorsteinsson E, Karpin A, Copeland J, Allsop D (2010). Computer-delivered interventions for alcohol and tobacco use: a meta-analysis. Addiction.

[44] Kodama S, Saito K, Tanaka S, Horikawa C, Fujiwara K, Hirasawa R, Yachi Y, Iida KT, Shimano H, Ohashi Y, Yamada N, Sone H (2012). Effect of web-based lifestyle modification on weight control: a meta-analysis. International Journal of Obesity.

[CR45] The pre-publication history for this paper can be accessed here:http://www.biomedcentral.com/1471-2458/14/809/prepub

